# The Empirical Bayes Variational Autoencoder—A Neural ODE Approach for Population Modeling in Pharmacology

**DOI:** 10.1002/psp4.70280

**Published:** 2026-06-17

**Authors:** Marcus Baaz, Anders Sjöberg, Mats Jirstrand

**Affiliations:** ^1^ Fraunhofer‐Chalmers Centre Gothenburg Sweden; ^2^ Department of Electrical Engineering Chalmers University of Technology Gothenburg Sweden

**Keywords:** empirical Bayes, neural ODEs, population modeling, Variational inference

## Abstract

Variational autoencoders (VAEs) combined with neural ordinary differential equations provide a flexible framework for exploring neural latent‐variable models in population pharmacokinetics. In this work, we investigate an empirical Bayes VAE formulation that integrates encoder–decoder architectures with covariate‐dependent population priors, enabling correlated latent representations and probabilistic inference. We evaluate the proposed framework using controlled simulation studies and a small clinical benchmark dataset. The simulation experiments assess the ability to recover known population structures and covariate effects, while the clinical study evaluates subject‐specific prediction and model diagnostics. In simulation studies with correlated individual parameters, the empirical Bayes VAE consistently captured population‐level variability, whereas a fixed‐prior VAE baseline exhibited systematic biases. In our experiments, extrapolation beyond the training dosing schedules showed more stable predictive behavior when using the proposed input–response normalization, relative to models trained without normalization, within a limited range. Diagnostic analyses indicated clear relationships between inferred latent variables and true parameters, and estimated observation noise was consistent with simulated values. In the clinical case study, cross‐validation experiments suggested predictive performance comparable to previously reported neural ODE–based approaches. Overall, the results illustrate the feasibility of combining empirical Bayes inference with neural ODE–based decoders for population modeling. The proposed framework should be viewed as a methodological proof‐of‐concept, highlighting both the potential and the current limitations of variational neural approaches in pharmacometric applications.

## Study Highlights

**What Is the Current Knowledge on the Topic?**

- Population modeling with nonlinear mixed‐effects models captures inter‐ and intra‐individual variability in drug response but is limited by rigid parametric assumptions.

**What Question Did This Study Address?**

- Can modern machine learning approaches—specifically variational autoencoders (VAEs) combined with neural ODEs and empirical Bayes priors—provide a flexible, scalable, and interpretable alternative to classical NLME models for population pharmacokinetics and pharmacodynamics?

**What Does This Study Add to Our Knowledge?**

- Empirical Bayes VAEs accurately capture individual variability, covariate effects, and nonlinear dynamics, offering a promising approach for exploring complex dynamics and automated model building.

**How Might This Change Drug Discovery, Development, and/or Therapeutics?**

- This approach offers a promising method for uncovering nonlinear and complex baseline covariate effects, enabling more accurate characterization of inter‐individual variability and enhanced integration of high‐dimensional biological data, complementing classical pharmacometric modeling.

## 1 Introduction

Population modeling is a cornerstone of pharmacometrics, providing a framework for quantifying inter‐ and intra‐individual variability in drug response [1]. Nonlinear mixed‐effects (NLME) models allow researchers to disentangle population‐level trends from subject‐specific dynamics, enabling robust parameter estimation, prediction, and extrapolation [2]. Traditionally, these models have been applied in drug development to describe pharmacokinetics (PK), pharmacodynamics (PD), and probabilistic events (time‐to‐event modeling) [3, 4, 5]. These approaches rely on strong parametric assumptions about structural models, variability distributions, and error models, which require substantial manual specification and iterative refinement, particularly when structural mechanisms or relevant covariates are uncertain.

Recent advances in machine learning (ML) have introduced neural ordinary differential equations (neural ODEs), which extend deep learning to continuous‐time dynamical systems [6]. Neural ODEs replace fixed parametric differential equations with neural networks that learn vector fields directly from data, thereby offering greater flexibility in modeling dynamics. In the context of biological and pharmacological systems, this provides a natural way to capture temporal evolution while accommodating nonlinearities and multivariate observations.

The integration of neural networks into population modeling has gained increasing attention [7, 8, 9, 10, 11, 12]. While small neural network structures have been implemented in established pharmacometric platforms such as Monolix [13], these implementations do not leverage backpropagation or automatic differentiation [10]. Consequently, training is computationally intensive and lacks scalability. As a result, the practical use of larger and more expressive architectures remains computationally challenging.

To address this limitation, autoencoder‐based approaches have been proposed [7, 8]. Autoencoders provide a flexible means of learning latent representations of individuals, which can be combined with neural ODEs to describe subject‐specific trajectories in continuous time. When formulated as variational autoencoders (VAEs) [14, 15, 16], these models further enable probabilistic inference over latent variables, bridging classical NLME modeling with modern deep learning.

Furthermore, formulating the VAE using an empirical Bayes approach allows the model to learn correlated population‐level latent effects and incorporate covariate information in a principled manner [17].

The aim of this study is threefold:

1. To illustrate the analogies between the stochastic approximation expectation–maximization (SAEM) algorithm, widely used in NLME modeling, and the VAE framework.
2. To investigate, using simulated data, the effect of an empirical Bayes prior on population‐level inference and recovery of correlated latent structures and baseline covariate effects relative to a fixed‐prior VAE baseline.
3. To model clinical PK data using the empirical Bayes VAE, and to compare its predictive performance on truncated data to that of a model from the literature [10].

## 2 Methods

### 2.1 Conceptual Overview

#### 2.1.1 From SAEM to Neural Latent‐Variable Models

The SAEM algorithm is one of the most used algorithms for parameter estimation in NLME models [18, 19]. By alternating between a stochastic estimation step, where individual random effects are sampled via Markov chain Monte Carlo (MCMC), and a maximization step, where population parameters are updated, SAEM provides robust estimates of both fixed effects and inter‐individual variability (IIV). This framework has been widely adopted in pharmacometrics and is implemented in major software such as Monolix and NONMEM [13, 20].

As datasets grow in complexity and incorporate richer sources of information, modeling approaches that can integrate heterogeneous inputs and utilize more flexible functional components, whilst remaining computationally tractable, become increasingly important. Neural networks, with their ability to learn nonlinear relationships and extract latent structure directly from data, offer a complementary modeling paradigm to the classical NLME methodology. However, the reliance on iterative MCMC sampling makes traditional SAEM approaches computationally intensive and less suitable for training large‐scale neural network models.

#### 2.1.2 The Variational Approach to Population Inference

In traditional NLME modeling, both the structural model—typically a fixed system of ODEs—and the form of the latent individual parameter distribution must be defined in advance. Our approach relaxes these assumptions by learning both the system dynamics and the distribution of individual‐specific parameters directly from data. We still consider a system of ODEs, but with the right‐hand side of it parametrized by a neural network, i.e., a neural ODE. Together with an additional network that maps the latent ODE states to the variable of interest, this constitutes what's known as a decoder (a model that reconstructs data). Another neural network, the encoder, approximates the latent individual parameters' posterior distribution. The posterior is assumed to be a Gaussian distribution; however, in combination with a flexible enough decoder, any parameter distribution that is a transformation of a Gaussian can, in theory, be modeled (given sufficiently large neural networks). This yields a flexible latent‐variable formulation that parallels classical NLME models while allowing more expressive functional components.

To illustrate the connection to classical population modeling, consider first the simplest estimation strategy: The naïve‐pooled approach, where all observations are assumed to originate from a single “hyper‐individual”. In a neural setting, this can be replicated using only the neural ODE decoder. To model IIV, an encoder can be introduced. A conventional autoencoder (AE) offers a neural analogue to the classical two‐stage approach in population modeling: The encoder infers subject‐specific parameters from individual data, and the neural ODE decoder reconstructs each subject's trajectory. However, just as the two‐stage method neglects the population structure, a standard autoencoder learns independent encodings for each individual, which can lead to poorly constrained parameters when data are sparse or noisy.

The VAE framework resolves this by introducing a population‐level prior, typically Gaussian, on the latent individual parameter posterior. This prior acts as a regularization mechanism, encouraging the encoder to produce a structured and continuous latent representation across the population. The result is a neural analogue to the NLME framework. An additional refinement is achieved through empirical Bayes estimation, in which the parameters of the population prior are not fixed but estimated directly from the data. Within the VAE framework, this allows the prior to adapt to the observed population rather than being constrained to a predefined distribution. This data‐driven prior learns parameter correlations and shapes the latent individual parameters to better reflect the underlying population structure. The resulting empirical Bayes VAE combines variational inference with population‐level regularization in a manner consistent with classical mixed‐effects principles.

### 2.2 Model Formulation

**FIGURE 1:**
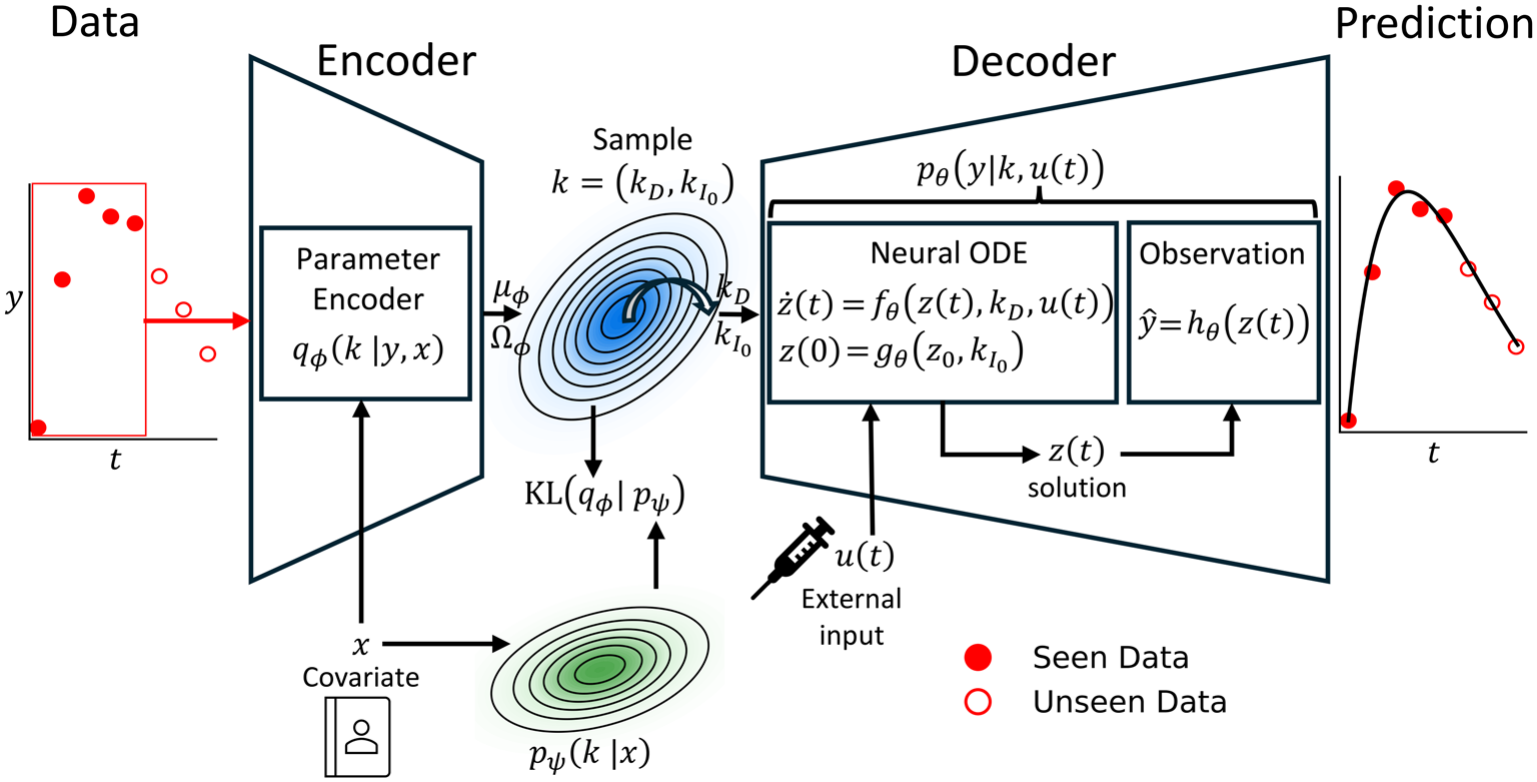
Overview of the empirical Bayes variational autoencoder. The encoder learns the posterior of the individual parameters k, which gives individual time course data and covariates. A sample is drawn from the posterior and sent to the decoder to facilitate reconstruction of each individual's data.

Our model, illustrated in Figure 1, follows an encoder–decoder architecture composed of a transformer‐based encoder and a neural ODE decoder with a Gaussian observation model [6, 21, 22]. The decoder combines neural ODE dynamics with a deterministic mapping to the concentration domain and a Gaussian additive–proportional residual error model. This formulation separates individual latent effects, latent dynamical states, structural dynamics, and measurement noise, providing a neural analogue to classical NLME modeling.

The subsequent sections introduce each component of the model in detail, including the representation of IIV, the latent dynamical system, the population prior, and a normalization strategy that disentangles external inputs from individual latent effects.

#### 2.2.1 Inter‐Individual Variability: Latent Representation and Encoder

For an individual j, observations are available at subject‐specific time points,

$$y^{\left(j\right)}=\left(y^{\left(j\right)}\left(t_{1}^{\left(j\right)}\right),\ldots ,y^{\left(j\right)}\left(t_{N_{j}}^{\left(j\right)}\right)\right),$$

where both $N_{j}$ and the sampling times $\left\{t_{i}^{\left(j\right)}\right\}$ may differ across individuals. Baseline covariates for individual j are denoted $x^{\left(j\right)}$. For notational clarity, we omit the superscript j in the model specification below.

IIV is represented by a latent vector,

$$k=\left(k_{I_{0}},k_{D}\right)\in R^{I_{0}+D},$$

where $k_{I_{0}}\in R^{I_{0}}$ encodes individual‐specific deviations in the initial condition of the neural ODE state, and $k_{D}\in R^{D}$ captures individual‐specific dynamic effects. The dimensions $I_{0}$ and D are treated as hyperparameters. The corresponding latent components $k_{I_{0}}$ and $k_{D}$ are inferred jointly, allowing arbitrary correlations between initial‐condition and dynamic effects.

Individual‐level posterior distributions over the latent individual parameters are approximated by an encoder,

$$\begin{array}{c} q_{\phi }\left(k|y,x\right)=N\left(\mu_{\phi }\left(y,x\right),\Omega_{\phi }\left(y,x\right)\right), \end{array}$$

$\text{which maps each}\;{\text{subject}}^{’}s\;\text{observed time series}\;y$ and baseline covariates x to the parameters of a multivariate Gaussian distribution. To ensure that $\Omega_{\phi }\left(y,x\right)$ is symmetric, positive definite, and numerically stable. It is internally parameterized via an unconstrained matrix $A_{\phi }\left(y,x\right)\in R^{\left(I_{0}+D\right)\times \left(I_{0}+D\right)}$ produced by the encoder:

$$\Omega_{\phi }\left(y,x\right)=A_{\phi }\left(y,x\right)\;A_{\phi }^{⊤}\left(y,x\right)+\mathrm{\epsilon I},\;\epsilon >0.$$

This construction guarantees that $\Omega_{\phi }\left(y,x\right)$ is a valid covariance matrix while keeping the encoder fully expressive. The quantity $\mu_{\phi }$ carries the same qualitative interpretation as the empirical Bayes estimates (EBEs) used in NLME modeling, i.e., the most likely individual parameter, with $\Omega_{\phi }$ describing the uncertainty.

The population prior for k is introduced later in the model formulation.

#### 2.2.2 Decoder: Neural ODE Dynamics, Observation Mapping, and Residual Error Model

Each individual may also receive a subject‐specific, time‐varying external input $u^{\left(j\right)}\left(t\right)$, e.g., a dosing schedule, which may include all external information available up to time t. As with $y^{\left(j\right)}$ and $x^{\left(j\right)}$. We omit the superscript j below for notational clarity.

The temporal evolution of the latent state $z\left(t\right)\in R^{M}$ is governed by a neural ODE. The vector field is parameterized by a neural network $f_{\theta }$, and individual‐specific deviations in the initial condition are incorporated through a mapping $g_{\theta }:$

$$\frac{\mathrm{dz}\left(t\right)}{\mathrm{dt}}=f_{\theta }\left(z\left(t\right),k_{D},u\left(t\right)\right),\;z\left(0\right)=g_{\theta }\left(z_{0},k_{I_{0}}\right),$$

where $z_{0}$ is a learnable population‐level initial state, and $g_{\theta }:R^{M+I_{0}}\to R^{M}$ lifts initial‐condition individual variability into the ODE state dimension.

While $z\left(t\right)$ may in some cases correspond directly to observed quantities, it generally evolves in a latent space that must be mapped to the measurement domain. We therefore introduce a deterministic observation mapping $h_{\theta }$, which projects the latent ODE state to the concentration space. In this work, $h_{\theta }$, is linear, ensuring that the nonlinear structure is captured by the neural ODE dynamics rather than absorbed by the observation mapping.

Observations are modeled using a classical additive–proportional residual error model.

$$y_{t_{i}}=h_{\theta }\left(z\left(t_{i}\right)\right)+\varepsilon_{i},\;\varepsilon_{i}\sim N\left(0,\sigma_{\mathrm{add}}^{2}+\sigma_{\text{prop}}^{2}h_{\theta }{\left(z\left(t_{i}\right)\right)}^{2}\right).$$

Here, $\sigma_{\mathrm{add}}$ represents constant additive noise and $\sigma_{\text{prop}}$ captures proportional noise that scales with the predicted observation, Together, the neural ODE dynamics, observation mapping, and noise model define the likelihood,

$$p_{\theta }\left(y|k,u\right),$$

where all decoder‐related parameters (including the population initial state $z_{0}$, the noise parameters $\sigma_{\mathrm{add}}$ and $\sigma_{\text{prop}}$, and the ODE/observation network weights) are absorbed into θ.

#### 2.2.3 Population Prior: Empirical Bayes Formulation

VAEs are typically formulated with a fixed standard normal prior $N\left(0,I\right)$. Instead, we adopt an empirical Bayes formulation in which the population prior is learned from data and allowed to depend on covariates. IIV is regularized through a covariate‐dependent multivariate Gaussian prior,

$$p_{\psi }\left(k|x\right)=N\left(\mu_{\psi }\left(x\right),\Omega_{\psi }\left(x\right)\right),$$

where $\mu_{\psi }\left(x\right)$ captures systematic covariate effects and $\Omega_{\psi }\left(x\right)$ represents population‐level variability. In an NLME formulation, these two quantities would correspond to covariate‐dependent fixed effects and the random‐effects omega matrix, respectively.

To maintain the same guarantees of symmetry, positive definiteness, and numerical stability as in the encoder, $\Omega_{\psi }\left(x\right)$ is parameterized analogously to the encoder covariance. This empirical Bayes prior adapts to the observed population while retaining a coherent probabilistic structure, yielding a covariate‐informed representation of IIV.

Combining the likelihood defined in the previous section with the population prior above yields the joint probabilistic model,

(1) $$\begin{array}{c} p_{\theta ,\psi }\left(y,k|x,u\right)=p_{\theta }\left(y|k,u\right)p_{\psi }\left(k|x\right). \end{array}$$

Note that this factorization relies on two conditional‐independence assumptions:

- k⊥u∣x, i.e., external inputs do not inform inter‐individual variability.
- $y⊥x|\left(k,u\right)$, i.e., covariates influence the observations only through the latent effects k.

#### 2.2.4 Input‐Response Normalization

Joint training of the encoder and decoder introduces a potential identifiability issue: Although the external input $u\left(t\right)$ is not provided directly to the encoder, its influence is embedded in the observed trajectories $y\left(t\right)$. Consequently, the encoder may attribute systematic input‐driven variation to the latent individual effects k, leading to confounding between structural dynamics and IIV. To mitigate this, training is performed in two stages.

**Decoder pretraining:**

The decoder is first trained with the latent effects fixed to the covariate‐dependent population mean, $k=\mu_{\psi }\left(x\right)$, effectively bypassing the encoder. Under this constraint, the neural ODE must account for input‐driven variation directly through the structural dynamics. This establishes a baseline input–response mapping before individual‐level variability is introduced.

**Input–response normalization:**

After pretraining, each individual's observed trajectory is divided by the decoder‐predicted response obtained under $k=\mu_{\psi }\left(x\right)$. This reduces systematic input‐driven structure in the encoder input, limiting the encoder's ability to attribute external input effects to the latent individual effects during subsequent variational training.

This procedure is designed to promote a clearer separation between input‐driven dynamics and latent IIV. It addresses an identifiability concern inherent to flexible encoder–decoder architectures and is not introduced as a mechanism for controlling model capacity. Conceptually, this normalization is akin to what is done in prediction‐corrected visual predictive checks (pcVPC), where observations (and simulations) are scaled relative to model predictions to remove systematic dose‐ or covariate‐driven effects [23].

## 3 Posterior Inference and Parameter Estimation

### 3.1 Bridging SAEM and VAEs Through the Posterior

Having introduced the joint probabilistic model in Equation (1), we now clarify the conceptual link between SAEM‐based population modeling and the variational formulation used in VAEs. Both approaches aim to maximize the marginal likelihood.

$$p_{\theta ,\psi }\left(y|u,x\right)=\int p_{\theta }\left(y|k,u\right)\;p_{\psi }\left(k|x\right)\;\mathrm{dk},$$

but differ in how they handle the intractable posterior over the individual effects k.

To relate the two, we introduce an auxiliary distribution $q\left(k|y,x\right)$ and use the standard variational identity

$$\begin{array}{c} \log p_{\theta ,\psi }\left(y|x,u\right)=\underset{=\text{ELBO}\left(\theta ,q,\psi \right)}{\underbrace{E_{q\left(k|y,x\right)}\left[\log p_{\theta ,\psi }\left(y,k|x,u\right)-\log q\left(k|y,x\right)\right]}}\\ \;+\mathrm{KL}\left(q\left(k|y,x\right)|p_{\theta }\left(k|y,x\right)\right), \end{array}$$

under the conditional‐independence assumption (a). Here, $\mathrm{KL}\left(\cdot |\cdot \right)$ denotes the Kullback–Leibler divergence and measures the discrepancy between the auxiliary distribution and the true posterior conditional on $\left(y,x\right)$. Since the KL divergence is non‐negative, the ELBO forms a valid lower bound on the marginal likelihood [24].

In the SAEM framework, the auxiliary distribution is set equal to the model posterior under the current parameter estimate $\theta_{r},\psi_{r}$ at iteration r,

$$q\left(k|y,x\right)∶=p_{\theta_{r},\psi_{r}}\left(k|y,x\right),$$

which reduces the ELBO to the classical EM Q‐function, $Q\left(\theta ,\psi |\theta_{r,}\psi_{r}\right)=E_{p_{\theta_{r},\psi_{r}}\left(k|y,x\right)}\left[\log p_{\theta ,\psi }\left(y,k|x,u\right)\right]$ [25]. Since the expectation generally cannot be computed analytically, SAEM approximates it by MCMC sampling of the latent variables, followed by a maximization step.

In the variational formulation, the auxiliary distribution is replaced by the previously presented encoder.

$$q\left(k|y,x\right)∶=q_{\phi }\left(k|y,x\right),$$

which provides an amortized, neural approximation to the true model posterior. Substituting this into the ELBO equation yields the VAE objective,

(2) $$\begin{array}{c} \text{ELBO}\left(\theta ,\phi ,\psi \right)=E_{q_{\phi }\left(k|y,x\right)}\left[\log p_{\theta }\left(y|k,u\right)\right]-\mathrm{KL}\left(q_{\phi }\left(k|y,x\right)|p_{\psi }\left(k|x\right)\right). \end{array}$$

The first term encourages the decoder to explain each individual's data through the latent effects k, while the KL term regularizes the encoder toward the population prior $p_{\psi }\left(k|x\right)$, thereby coupling individual and population levels in the variational approximation.

### 3.2 Practical Training Considerations

Training proceeds by maximizing the ELBO in Equation (2) jointly over all model parameters $\left(\theta ,\phi ,\psi \right)$ using minibatch gradient‐based stochastic optimization [26]. For a minibatch B of individuals, the objective is,

$$\max_{\theta ,\phi ,\psi }\frac{1}{\left|B\right|}\sum_{j\in B}{\text{ELBO}}^{\left(j\right)}\left(\theta ,\phi ,\psi \right),$$

where ${\text{ELBO}}^{\left(j\right)}$ denotes the contribution of the individual j.

Gradients with respect to the encoder parameters ϕ are computed using the reparameterization trick [16]: Latent individual parameters are expressed as,

$$k=\mu_{\phi }\left(y,x\right)+L_{\phi }\left(y,x\right)\;\epsilon ,\;\epsilon ∼N\left(0,I\right),$$

where $L_{\phi }\left(y,x\right)$ is the Cholesky factor of the covariance matrix, i.e., $L_{\phi }\left(y,x\right)L_{\phi }^{⊤}\left(y,x\right)=\Omega_{\phi }\left(y,x\right)$ [27]. Expectations are approximated using S Monte Carlo samples per individual. The choice of S is a hyperparameter that balances estimator variance and computational cost.

Gradients through the neural ODE decoder are obtained by differentiating the fixed‐step Runge–Kutta solver (used for integrating the latent neural states) directly; since the latent trajectories in our examples are short, the adjoint sensitivity method [6] is unnecessary, and direct backpropagation yields stable and accurate gradients at moderate memory cost.

Under the Gaussian additive and proportional noise assumptions, the reconstruction term in the ELBO corresponds to a weighted mean‐squared error, with weights determined by the learned noise parameters $\sigma_{\mathrm{add}}$, $\sigma_{\text{prop}}$, and the model predictions.

Finally, training is preceded by the pretraining phase described in the *Input–Response Normalization* section. This phase uses an $L^{1}$‐norm loss to obtain a median‐based normalization.

## 4 Experiments

The two experimental settings were selected to provide complementary validation of the proposed framework. The simulation study was conducted under controlled conditions with a large number of individuals to assess identifiability and recovery of known population structure, while limiting confounding effects due to limited sampling. In contrast, the clinical case study is based on a small‐sample benchmark previously studied using low‐dimensional neural ODEs, which were shown to achieve predictive performance comparable to classical compartment models, with modest improvements in some settings [10]. This provides a relevant reference point for evaluating practical feasibility in data‐limited scenarios.

In all experiments, the posterior dimensions were set to $I_{0}=1$ and D=2, and the latent decoder dimensions to M=2. They were deliberately kept low to impose a strong information bottleneck and promote regularization. These values were chosen based on the expected dimensionality of the underlying PK processes and standard model diagnostics. When faced with a new dataset, we envision two complementary strategies for determining the latent dimensionality. One is a model‐driven approach, where pharmacometric knowledge—such as typical compartment structures and expected sources of IIV—is used to define a plausible latent dimension. The other is a data‐driven approach, where models with different latent dimensions are compared using predictive diagnostics or sensitivity analyses.

The decoder networks contained around 136,000 and 500,000 parameters $\left(\theta \right)$ in the case study and simulation study, respectively. In both cases, the encoder contained approximately 900,000 parameters $\left(\phi \right)$, while the prior network contained 140 parameters $\left(\psi \right)$. Although the encoder and decoder are highly overparameterized, the amount of information that can be transmitted from the encoder to the neural ODE—and subsequently from the neural ODE to the observation model—is strongly constrained by the small dimensionality of the latent individual parameters and the latent ODE. This information bottleneck limits the model's capacity to overfit. We also tested substantially smaller networks (θ=2700,ϕ=34000), which produced similar diagnostic plots (see DATA S4). All neural network models were implemented in Python.

### 4.1 The Simulation Study

To evaluate the models under controlled conditions, we generated synthetic pharmacokinetic data from a standard one‐compartment model, under a repeated‐dosing schedule, with first‐order absorption. IIV was introduced via random effects on the initial concentration ($C_{0}$), absorption rate constant ($k_{a}$), and the elimination rate constant ($k_{e}$). The initial concentration was not dose‐dependent and could be the result of a run‐in period prior to the simulated dosing regimen. All values were sampled from a multivariate normal distribution with a full covariance matrix. In the first experiment, one training set and two test sets were created, with one test set sharing the same treatment schedule as the training set. This experiment aimed to evaluate the ability of the fixed‐prior VAE and empirical Bayes VAE to model data where individual parameters were simulated with correlation. Furthermore, the fixed‐prior VAE was trained without the input‐response normalization on a dataset simulated without correlated individual parameters, to show why the procedure is necessary.

For the second experiment, data were generated with a baseline covariate effect on $k_{e}$. Here, test data was only generated with the same schedule as the training data, and only the empirical Bayes VAE was evaluated. In both experiments, the models were trained until they could describe the training data on a population level, then predictions on the test sets were performed. See DATA S1 and DATA S3 for more information regarding the model parameters and experimental setup.

### 4.2 Case Study

To evaluate whether the model could be trained on small datasets, we trained the model using a *clinical* benchmark dataset [13]. Patients received a single oral dose of theophylline, and plasma concentration measurements were taken up to approximately 24 h post‐dose.

To assess predictive performance under partial observation, we withheld the last three observation time points from the encoder and evaluated predictions for these time points on held‐out individuals. A related experiment was reported by Bräm et al. [10]., where a low‐dimensional neural ODE was implemented in Monolix [13] and trained using SAEM. That study reported predictive performance broadly comparable to classical NLME models, with modest improvements in some settings, including the theophylline benchmark, and slightly worse performance in others.

We performed 100 random 70/30 cross‐validation splits, with 70% of the individuals used for training and 30% held out for testing. For the Bräm model, estimations were performed in Monolix. All implementations were done in accordance with the code and instructions provided in the original paper. For the Bräm model, the test individuals were kept in the training loop, with their last three measurements removed to easily obtain the EBEs, whereas for the empirical Bayes VAE case, they were not included in the training at all.

## 5 Results

**FIGURE 2:**
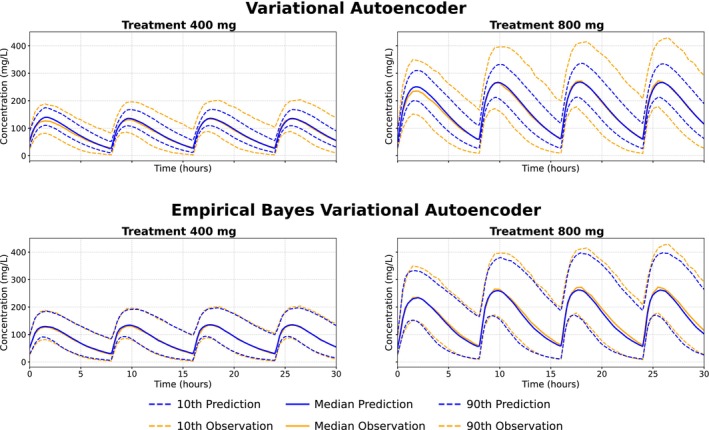
The top row shows population model simulations (blue) from the fixed‐prior variational autoencoder, based on sampling from the prior, compared to observed data (orange). The bottom row shows the same comparison for the empirical Bayes variational autoencoder.

All population simulations were performed by drawing samples from the prior with the same size used during training. For data generated with correlated individual parameters, the empirical Bayes VAE reproduced the population distribution accurately, whereas the fixed‐prior VAE failed to capture the correct variability when sampling from a fixed standard Gaussian prior (Figure 2).

**FIGURE 3:**
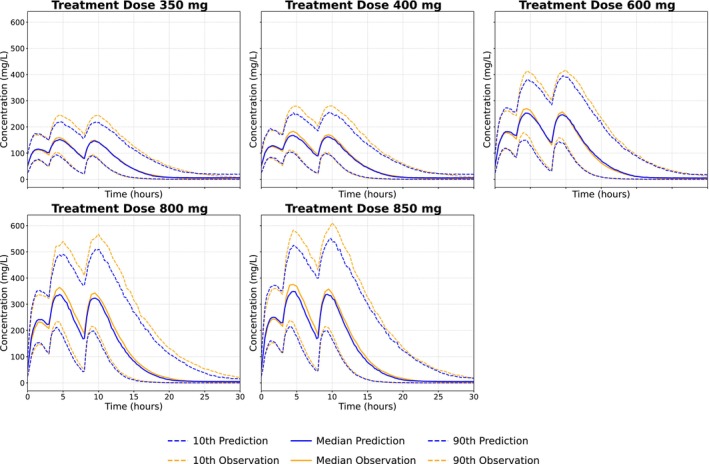
Model predictions (blue) evaluation using the empirical Bayes variational autoencoder trained on the 400‐mg and 800‐mg dosing schedules (days 0, 8, 16, and 24), with evaluations performed on dosing regimens of 350, 400, 600, 800, and 850 mg administered on days 0, 3, and 8.

**FIGURE 4:**
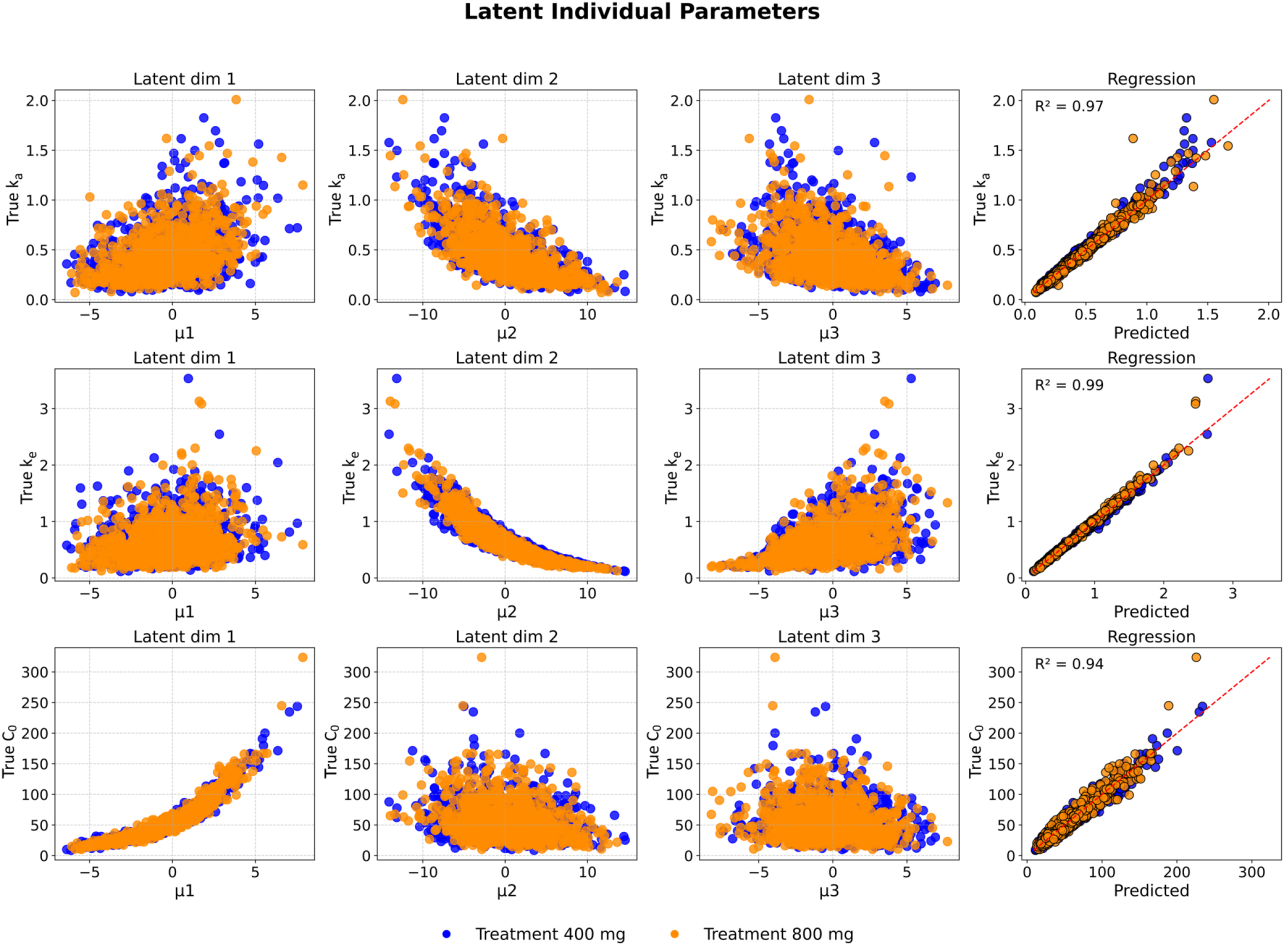
The first three columns display the true model parameters alongside the prior‐normalized mean posterior estimates for each individual, with treatment arms distinguished by color. The final column presents predictions from a random forest regression model, trained on the training set, applied to prior‐normalized posterior means from the test set.

When evaluated on dosing schedules not seen during training, the empirical Bayes VAE maintained strong predictive performance across dose levels and intervals (Figure 3). Minor discrepancies were observed only at the upper percentiles of the highest doses. Figure 4 summarizes the relationship between the true simulated parameters and the posterior means inferred by the empirical Bayes VAE. The first three columns show a clear correspondence between prior‐normalized posterior means and ground‐truth values. To further assess identifiability, a Random Forest model was trained on the prior‐normalized posterior means from the training set and used to predict individual parameters in the test set; these predictions are visualized in the final column [28]. See DATA S2 for the same plots for the case when the fixed‐prior VAE was trained without the input‐response normalization. The encoder entangles dose with the individual parameters, inducing clustering in the posterior means and, in turn, causing substantial bias in population predictions for both dosing schedules.

**FIGURE 5:**
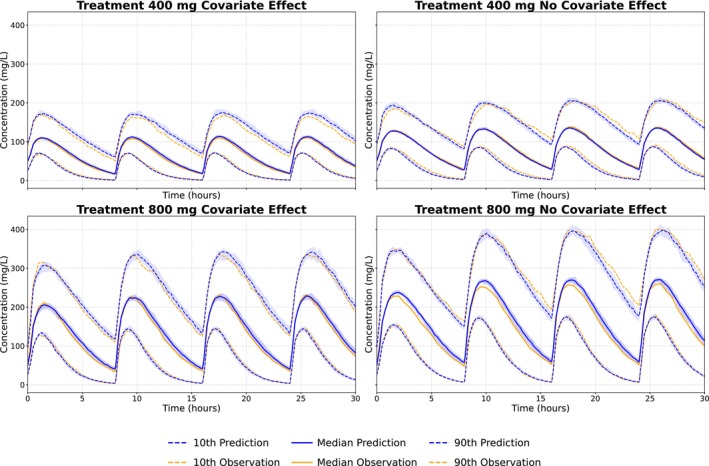
Empirical Bayes variational autoencoder simulations compared to test data stratified on both treatment arm and covariate. The 95% confidence interval, based on 10 simulations, is shown as shaded areas.

Figure 5 presents results from the scenario including both a covariate effect on the elimination rate and full covariance among individual parameters. The empirical Bayes VAE recovered the covariate effect and correlation structure, and the estimated additive and proportional error standard deviations deviated by less than 1% from their true values, indicating accurate uncertainty quantification. Overall, the model showed strong agreement with the simulated data.

**FIGURE 6:**
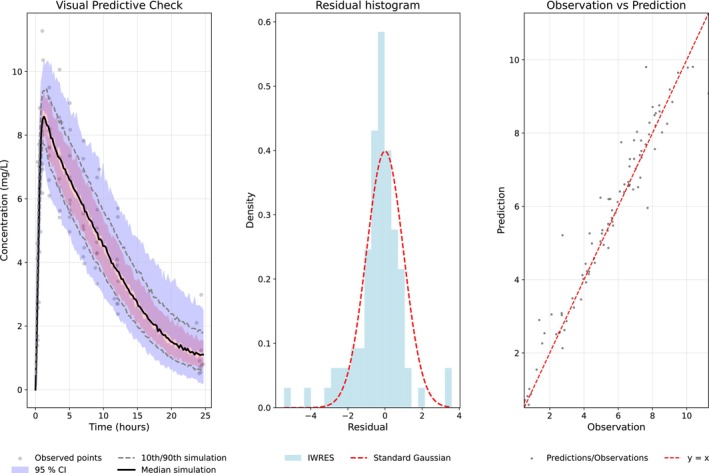
Visual predictive check, histogram of individual weighted residuals (IWRES), and observed concentrations plotted against model predictions from an empirical Bayes variational autoencoder trained on the clinical dataset.

**FIGURE 7:**
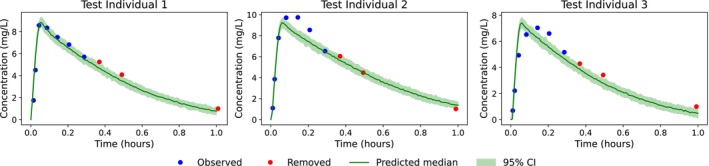
Individual predictions on the clinical test dataset with associated 95% prediction confidence intervals. Blue points indicate samples used by the encoder, while red points correspond to removed data.

For the clinical case study, model diagnostics—including individual weighted residuals, observed vs. predicted concentrations, and visual predictive checks (Figure 6)—indicated an overall satisfactory fit. Predictions for one representative cross‐validation split are shown in Figure 7.

Across the 100 random 70/30 cross‐validation splits, predictive performance was summarized using the median absolute prediction error computed on held‐out individuals. The empirical Bayes VAE achieved a median value of 0.42 compared to 0.57 for the Bräm model. Because a small proportion of splits for the Bräm model exhibited substantially higher error, we additionally evaluated trimmed performance by excluding the worst 5% of prediction errors for each method. Under this condition, the trimmed mean squared error (MSE) was 0.40 for the empirical Bayes VAE and 0.46 for the Bräm model, with corresponding trimmed R^2^ values of 0.88 and 0.87. All metrics were computed exclusively on withheld test individuals.

## 6 Discussion

VAEs combined with neural ODEs are conceptually similar to classical NLME models. The encoder extracts latent representations capturing individual variability, analogous to random effects (Figure 3), while the decoder reconstructs observed trajectories through the learned dynamical system. Unlike SAEM, which relies on iterative, sampling‐based estimation, the VAE uses gradient‐based optimization, allowing for more scalable and efficient inference as the number of individuals or, more importantly, model parameters increases. In addition, the model is agnostic to data modality and covariate structure, enabling the integration of diverse feature types within a unified and coherent inference framework. Moreover, compared with an AE, the VAE provides a probabilistic latent representation that quantifies uncertainty and IIV and supports prediction in new populations.

At first glance, the model may seem heavily overparameterized relative to the dataset size. However, although the encoder and decoder contain a large number of parameters, these should not be interpreted as classical “independent” model parameters. Instead, one important measure of effective model complexity—particularly in relation to pharmacometric models—is the dimensionality of the latent space, while the remaining network parameters primarily define a flexible functional mapping learned through optimization.

Neural networks in general exhibit strong parameter dependencies, structural constraints, and implicit regularization, such that their effective model complexity can be substantially lower than what raw parameter count alone may suggest [29, 30]. In VAEs, this is further reinforced by the low‐dimensional latent bottleneck and KL regularization.

The motivation for the empirical Bayes formulation arises from limitations of a fixed standard normal prior as a parameterization for population modeling. While correlated dependencies may in principle be represented through the learned encoder–decoder mapping, a diagonal non‐trainable prior does not encode these explicitly, which can make learning such structure more difficult in practice. Introducing trainable off‐diagonal covariance elements allows the model to represent such correlations explicitly in the prior. Allowing the diagonal elements to be learned enables the prior to better match observed variance across individuals, which can further improve the latent parameterization. Conditioning the prior on covariates provides a principled mechanism for incorporating known sources of variability directly into the latent space, allowing the population distribution to adapt to patient‐specific information and supporting the modeling of potentially nonlinear covariate effects and interactions. Importantly, these extensions do not modify the loss function: The model continues to optimize the exact ELBO. As a result, uncertainty estimates follow directly from the variational objective and remain fully data‐driven, in contrast to approaches that alter the objective function by introducing weighting schemes or additional penalty terms [31].

Another critical consideration is the disentanglement of external input and baseline covariates from the latent individual effects. Simply providing them alongside observations does not guarantee that this mapping is identified. Our input‐response normalization strategy mitigates this by pretraining the decoder to fully leverage the external inputs. In combination with the covariate‐conditioned prior, this can reduce bias in population‐level predictions, since latent individual effects are sampled from the prior. Individual‐level inference requires the encoder to learn covariate–latent relationships. To address this, we propose a potential solution: A post‐training step in which each individual's latent parameters are normalized using the covariate‐conditioned prior, after which the encoder is retrained to predict these normalized parameters from the data. This encourages the model to focus on unexplained variability, rather than absorbing systematic covariate effects. However, since the experiment we perform with individual prediction (on the clinical data) doesn't include differences in external inputs or covariates, unbiased predictions are obtained without this, as can be seen in Figure 6. Therefore, a detailed exploration of this procedure is beyond the scope of this paper. More broadly, promoting independence between latent effects and known variability mirrors the classical principle that empirical Bayes estimates should not depend on treatment or baseline covariates.

The results indicate that the empirical Bayes VAE performed well across both simulated and clinical applications. The model demonstrated stable predictive behavior under moderate shifts in dosing schedules and incorporated covariate effects naturally through the covariate‐conditioned prior. In the simulation study, the use of a large dataset with substantial additive and proportional measurement error and IIV ensured that predictive uncertainty was driven primarily by aleatoric variability rather than epistemic uncertainty due to limited information. The results from the truncation experiment on the clinical dataset are consistent with previous reports, although the small sample size and variability across cross‐validation splits make it difficult to draw firm conclusions regarding comparative performance. Being more scalable, we expect our method to yield improved results with larger datasets.

It is important to distinguish between interpolation within observed dosing regimes and extrapolation far beyond the available data. Classical pharmacometric models can incorporate mechanistic saturation effects through predefined nonlinear structures, such as Michaelis–Menten or Imax relationships, which enable extrapolation when supported by appropriate data. In contrast, neural ODE–based models primarily rely on data‐driven regularization and may have limited ability to recover such asymptotic behavior in sparsely observed regions. Consequently, the extrapolation performance demonstrated in this study should be interpreted as generalization within the empirical dose–time domain rather than as prediction in previously unobserved pharmacological regimes.

In summary, the empirical Bayes VAE offers a powerful complement to classical population modeling techniques. By combining encoder–decoder architectures with empirical Bayes estimation, it provides a scalable, structured, and robust framework for capturing IIV in pharmacological systems, particularly in contexts where traditional parametric assumptions may be limiting. More broadly, this approach illustrates how machine learning can augment, rather than replace, mechanistic and statistical models. For instance, joint‐modeling frameworks could retain a mechanistic pharmacodynamic model to describe longitudinal dynamics, while parameterizing the hazard function that governs survival outcomes with a neural network. In this setup, the mechanistic model captures well‐established biological processes, whereas the neural network flexibly learns complex risk relationships that may be difficult to specify mechanistically. These hybrid strategies leverage the strengths of both classical modeling and modern machine learning, enabling richer, more flexible, and biologically grounded population inference.

## Author Contributions

M.B., A.S., and M.J. wrote the manuscript; M.B. and A.S. designed the research; M.B. and A.S. performed the research; M.B. and A.S. analyzed the data.

## Funding

No funding was received for this work.

## Conflicts of Interest

The authors declare no conflicts of interest.

## Supporting information


**Data S1:** Network Overview.

**Data S2:** Results without input‐response normalization.

**Data S3:** Simulation setup.

**Data S4:** Results with smaller networks.

## Data Availability

A detailed description of the model architecture, ODE solver configuration, encoder and decoder networks, and the full training procedure is provided in the DATA S1. All code used in this work, including model implementation, simulation scripts, and analysis notebooks, is available in our public repository https://github.com/FraunhoferChalmersCentre/neuralODEpopulation. The clinical data analyzed are available directly from Monolix, and the Bräm model is available from their paper.

## References

[1] B. Ribba , N. H. Holford , P. Magni , et al., “A Review of Mixed‐Effects Models of Tumor Growth and Effects of Anticancer Drug Treatment Used in Population Analysis,” CPT: Pharmacometrics & Systems Pharmacology 3, no. 5 (2014): e113, 10.1038/psp.2014.12. 24806032 PMC4050233

[2] M. J. Lindstrom and D. M. Bates , “Nonlinear Mixed Effects Models for Repeated Measures Data,” Biometrics 46, no. 3 (1990): 673–687, 10.2307/2532087. 2242409

[3] C. Mbogning , K. Bleakley , and M. Lavielle , “Joint Modelling of Longitudinal and Repeated Time‐To‐Event Data Using Nonlinear Mixed‐Effects Models and the Stochastic Approximation Expectation–Maximization Algorithm,” Journal of Statistical Computation and Simulation 85, no. 8 (2015): 1512–1528, 10.1080/00949655.2013.878938.

[4] A. Lindauer , C. R. Valiathan , K. Mehta , et al., “Translational Pharmacokinetic/Pharmacodynamic Modeling of Tumor Growth Inhibition Supports Dose‐Range Selection of the Anti–PD‐1 Antibody Pembrolizumab,” CPT: Pharmacometrics & Systems Pharmacology 6, no. 1 (2017): 11–20, 10.1002/psp4.12130. 27863176 PMC5270293

[5] L. Claret , P. Girard , P. M. Hoff , et al., “Model‐Based Prediction of Phase III Overall Survival in Colorectal Cancer on the Basis of Phase II Tumor Dynamics,” Journal of Clinical Oncology 27, no. 25 (2009): 4103–4108, 10.1200/JCO.2008.21.0807. 19636014

[6] R. T. Q. Chen , Y. Rubanova , J. Bettencourt , and D. Duvenaud , “Neural Ordinary Differential Equations [Internet],” arXiv (2019), 10.48550/arXiv.1806.07366.

[7] J. Lu , K. Deng , X. Zhang , G. Liu , and Y. Guan , “Neural‐ODE for Pharmacokinetics Modeling and Its Advantage to Alternative Machine Learning Models in Predicting New Dosing Regimens,” iScience 24, no. 7 (2021): 102804, 10.1016/j.isci.2021.102804. 34308294 PMC8283337

[8] M. Laurie and J. Lu , “Explainable Deep Learning for Tumor Dynamic Modeling and Overall Survival Prediction Using Neural‐ODE,” npj Systems Biology and Applications 9, no. 1 (2023): 58, 10.1038/s41540-023-00317-1. 37980358 PMC10657412

[9] D. S. Bräm , N. Parrott , L. Hutchinson , and B. Steiert , “Introduction of an Artificial Neural Network–Based Method for Concentration‐Time Predictions,” CPT: Pharmacometrics & Systems Pharmacology 11, no. 6 (2022): 745–754, 10.1002/psp4.12786. 35582964 PMC9197537

[10] D. S. Bräm , B. Steiert , M. Pfister , B. Steffens , and G. Koch , “Low‐Dimensional Neural Ordinary Differential Equations Accounting for Inter‐Individual Variability Implemented in Monolix and NONMEM,” CPT: Pharmacometrics & Systems Pharmacology 14, no. 1 (2025): 5–16, 10.1002/psp4.13265. 39552211 PMC11706426

[11] Z. Wu , P. Luo , R. Chen , Y. Liu , W. Jian , and T. Zhou , “Neural Controlled Differential Equation and Its Application in Pharmacokinetics and Pharmacodynamics,” CPT: Pharmacometrics & Systems Pharmacology 15, no. 1 (2026): e70146, 10.1002/psp4.70146. 41241764 PMC12823316

[12] I. B. Losada and N. Terranova , “Bridging Pharmacology and Neural Networks: A Deep Dive Into Neural Ordinary Differential Equations,” CPT: Pharmacometrics & Systems Pharmacology 13, no. 8 (2024): 1289–1296, 10.1002/psp4.13149. 38992975 PMC11330178

[13] “Monolix 2021R2, Lixoft SAS, a Simulations Plus company”.

[14] D. P. Kingma and M. Welling , “An Introduction to Variational Autoencoders,” Foundations and Trends in Machine Learning 12, no. 4 (2019): 307–392, 10.1561/2200000056.

[15] J. Rohleff , F. Bachmann , U. Nahum , et al., “Redefining Parameter Estimation and Covariate Selection via Variational Autoencoders: One Run Is All You Need,” CPT: Pharmacometrics & Systems Pharmacology 14, no. 12 (2025): 2232–2243, 10.1002/psp4.70129. 41186137 PMC12706427

[16] D. P. Kingma and M. Welling , “Auto‐Encoding Variational Bayes,” arXiv (2022), 10.48550/arXiv.1312.6114.

[17] W. Cheng , G. Darnell , S. Ramachandran , and L. Crawford , “Generalizing Variational Autoencoders With Hierarchical Empirical Bayes,” arXiv (2020), 10.48550/arXiv.2007.10389.

[18] B. Karimi , M. Lavielle , and E. Moulines , “F‐SAEM: A Fast Stochastic Approximation of the EM Algorithm for Nonlinear Mixed Effects Models,” arXiv (2019), 10.48550/arXiv.1910.12222.

[19] M. Lavielle and C. Mbogning , “An Improved SAEM Algorithm for Maximum Likelihood Estimation in Mixtures of Non Linear Mixed Effects Models,” Statistics and Computing 24, no. 5 (2014): 693–707, 10.1007/s11222-013-9396-2.

[20] NONMEM Project Group , “NONMEM”.

[21] A. Vaswani , N. Shazeer , N. Parmar , et al., “Attention Is All You Need,” arXiv (2023), 10.48550/arXiv.1706.03762.

[22] K. Aitken , V. V. Ramasesh , Y. Cao , and N. Maheswaranathan , “Understanding How Encoder‐Decoder Architectures Attend,” arXiv (2021), 10.48550/arXiv.2110.15253.

[23] M. Bergstrand , A. C. Hooker , J. E. Wallin , and M. O. Karlsson , “Prediction‐Corrected Visual Predictive Checks for Diagnosing Nonlinear Mixed‐Effects Models,” AAPS Journal 13, no. 2 (2011): 143–151, 10.1208/s12248-011-9255-z. 21302010 PMC3085712

[24] C. M. Bishop , Pattern Recognition and Machine Learning (Springer, 2006).

[25] A. P. Dempster , N. M. Laird , and D. B. Rubin , “Maximum Likelihood From Incomplete Data via the EM Algorithm,” Journal of the Royal Statistical Society. Series B, Statistical Methodology 39, no. 1 (1977): 1–22, 10.1111/j.2517-6161.1977.tb01600.x.

[26] R. M. Gower , N. Loizou , X. Qian , A. Sailanbayev , E. Shulgin , and P. Richtarik , “SGD: General Analysis and Improved Rates,” arXiv (2019), 10.48550/arXiv.1901.09401.

[27] P. K. Vishwakarma , “Cholesky Decomposition for Symmetric Matrices Over Finite Fields,” arXiv (2025), 10.48550/arXiv.2508.04657.

[28] L. Breiman , “Random Forests,” Machine Learning 45, no. 1 (2001): 5–32, 10.1023/A:1010933404324.

[29] M. Belkin , D. Hsu , S. Ma , and S. Mandal , “Reconciling Modern Machine Learning Practice and the Bias‐Variance Trade‐Off,” Proceedings of the National Academy of Sciences 116, no. 32 (2019): 15849–15854, 10.1073/pnas.1903070116. PMC6689936 31341078

[30] Z. Allen‐Zhu , Y. Li , and Y. Liang , “Learning and Generalization in Overparameterized Neural Networks, Going Beyond Two Layers,” arXiv (2020), 10.48550/arXiv.1811.04918.

[31] M. Fil , M. Mesinovic , M. Morris , and J. Wildberger , “Beta‐VAE Reproducibility: Challenges and Extensions,” arXiv (2021), 10.48550/arXiv.2112.14278.

