# Supplementary material for: The Empirical Bayes Variational Autoencoder—A Neural ODE Approach for Population Modeling in Pharmacology
Source: CPT Pharmacometrics Syst Pharmacol. 2026 Jun 17;15(7):e70280. doi: 10.1002/psp4.70280 (PMC13275335; doi:10.1002/psp4.70280)
Supplement: Supplementary file 1 — Data S1: Network Overview. [file PSP4-15-e70280-s002.docx]

## Network Overview

**Decoder**

The neural ODE consists of a deep fully connected network with four layers and SELU activations between them. It has a hidden dimension of 512 for the simulated data case and 256 for the clinical data case and includes a skip connection from the input to the output to improve gradient flow. The latent state, dynamic parameters, and drug representation are concatenated and fed into this network to compute the derivative of the latent state. Drug doses are represented as Dirac delta pulses over time. These dose pulses are passed through a smaller fully connected network with two layers and SELU activation, mapping the drug input dimension to a hidden dimension and back to the original drug dimension. The outputs of the dose network are combined with the latent input and processed by the main ODE network. The final derivative is obtained by summing the output of the main network and the skip connection. A trainable scalar controls the width of the Gaussian pulses used to model the doses.

The Observation model maps the latent state to the observed output with a single linear layer, taking the latent vector and producing a scalar output.

**Encoder**

The transformer-based encoder is designed to produce a posterior distribution over the parameters of the neural ODE. The input, consisting of time and observed states, is first projected into a hidden dimension of size 36 using a two-layer fully connected network with SELU activations. Positional encodings of size 128 are added to retain temporal information.

The transformer backbone has 3 layers of multi-head self-attention with 4 heads per layer. Each layer includes layer normalization, dropout with rate 0.001, and a feedforward network with SELU activations. The transformer outputs are pooled using multi-head attention pooling, producing a fixed-size representation which is then normalized.

The posterior mean is computed from the pooled representation via two fully connected layers mapping to the total number of parameters (sum of IC and dynamic parameters). The posterior covariance is computed through two fully connected layers producing a flattened matrix, reshaped to a square matrix, and combined to form a Cholesky factor for sampling.

The median Initial Conditions (IC) for the ODE is either a learnable parameter (if external input-independent) or computed via a two-layer fully connected network with SELU activations taking the first dose as input (if external input-dependent). The IC is further transformed using another two-layer fully connected network with SELU activations that combines the median IC with individual-specific initial condition parameters.

The encoder supports learning the prior mean and covariance, conditioned on baseline covariates, with the prior covariance optionally full or diagonal. It also supports multiple Monte Carlo samples for the posterior or prior.

**Training Procedure Overview**

The model is trained using a combination of neural ODEs, a transformer-based encoder, and a decoder network with trainable noise. Training follows a standard supervised framework with reconstruction and KL divergence losses, while accommodating optional autoencoder (AE) or variational autoencoder (VAE) modes.

**Initialization:**
Before training begins, the model components (ODEFunc, encoder, decoder, and noise module) are initialized on the appropriate device. Key hyperparameters such as latent dimension, parameter encoder dimension, batch size, and dense time grids are set. Initial metrics for validation mean squared error (MSE), log-likelihood (LL), and total loss are established, along with early stopping counters. Epoch-level metrics, including accumulated loss, KL divergence, reconstruction loss, MSE, latent vectors, and trajectory records, are reset at the start of each epoch.

**Data Handling:**
Measurements are z-normalized, time and dose are normalized with their maximum values. Observations and dose times are preprocessed into padded tensors with corresponding masks. Differentiable linear interpolation is applied to map sparse observations onto dense time grids, ensuring compatibility with ODE solvers. Covariates and dose-dependent effects are handled within the encoder and ODE input preparation.

**Forward Pass and Loss Computation:**
For each batch (5% of total data), the encoder produces posterior distributions over latent variables (mean and Cholesky covariance). If batch size is lower than 50 samples, additional samples are drawn. Initial conditions for the ODE are computed, incorporating both dose-independent and dose-dependent parameters as appropriate. The ODE is then integrated using RK4, and the decoder reconstructs observations over the dense time grid.

The loss function combines:

- A reconstruction loss accounting for additive and proportional observation noise
- A KL divergence term between the posterior and prior latent distributions, optionally using free bits and KL warm-up strategies

Metrics such as MSE and negative log-likelihood are tracked per batch and accumulated over the epoch.

**Backpropagation and Optimization:**
Gradients are computed via standard backpropagation using the ADAM optimizer. Gradient clipping is applied to stabilize training, and optimizer steps update trainable parameters. Learning rates are scheduled based on epoch-level performance metrics, with early stopping triggered if validation metrics fail to improve over a predefined patience period.

**Noise and EMA Updates:**
The noise module parameters are optionally frozen for initial warm-up epochs. After the warm-up period, additive and proportional noise parameters are updated along with the rest of the model. Exponential moving averages (EMA) are applied to all model components starting at a specified epoch for smoother parameter estimates.

**Validation and Early Stopping:**
Validation is performed periodically by computing predictions over the validation dataset, including MSE, LL, and total loss. The best model state is saved based on validation performance. Early stopping monitors MSE or log-likelihood depending on whether the model operates in AE or VAE mode, restoring the best model state if training is halted.

**Logging:**
At the end of each epoch, key statistics are printed, including epoch duration, MSE, loss components, KL divergence, additive noise, and learning rate. Optional validation metrics are also logged.
