# Supplementary material for: The Empirical Bayes Variational Autoencoder—A Neural ODE Approach for Population Modeling in Pharmacology
Source: CPT Pharmacometrics Syst Pharmacol. 2026 Jun 17;15(7):e70280. doi: 10.1002/psp4.70280 (PMC13275335; doi:10.1002/psp4.70280)
Supplement: Supplementary file 2 — Data S2: Results without input‐response normalization. [file PSP4-15-e70280-s001.docx]

## Results without input-response normalization


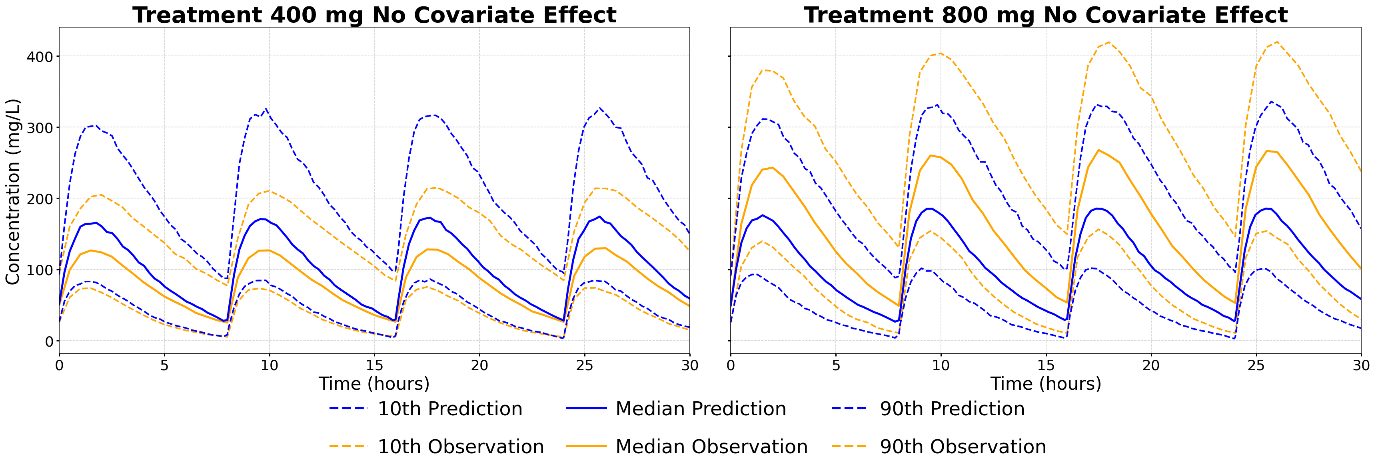


Figure A1. Model predictions (blue) evaluation using the variational autoencoder without input-response normalization.


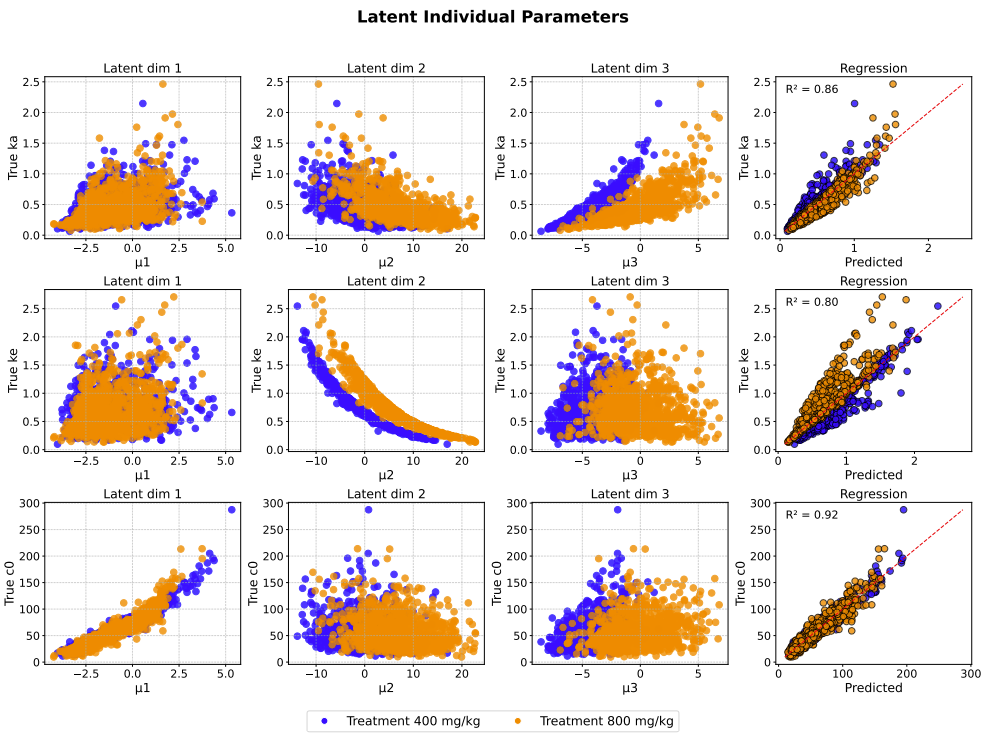


Figure A2. The first three columns display the true model parameters alongside the mean posterior estimates for each individual, with treatment arms distinguished by color. The final column presents predictions from a random forest regression model, trained on the training set, applied to posterior means from the test set. This was using the variational autoencoder without input-response normalization.


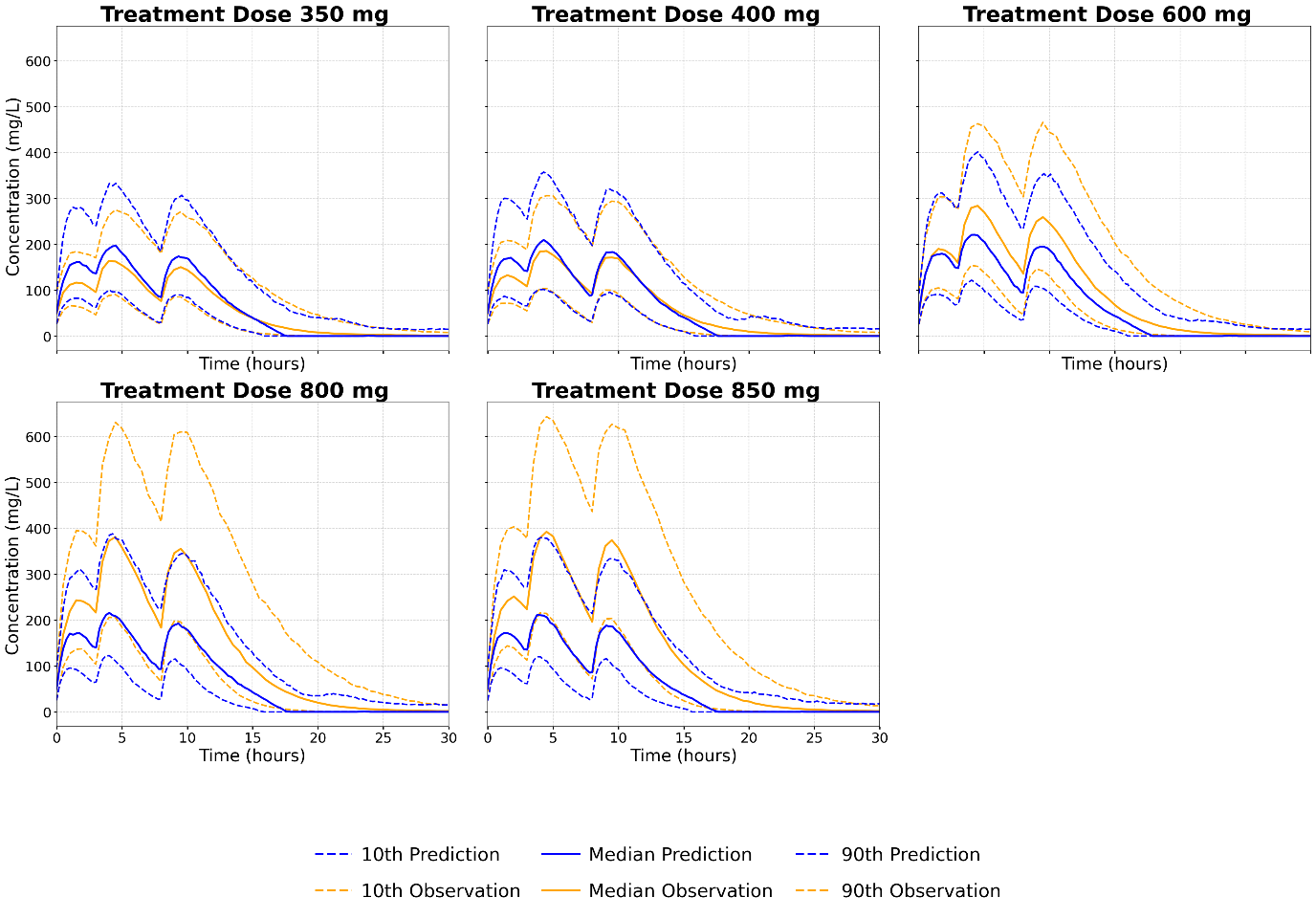


*Figure A3. Model predictions (blue) evaluation using the variational autoencoder without input-response normalization trained on the 400-mg and 800-mg dosing schedules (days 0, 8, and 16), with evaluations performed on dosing regimens of 350, 400, 600, 800, and 850 mg administered on days 0, 3 and 8.*


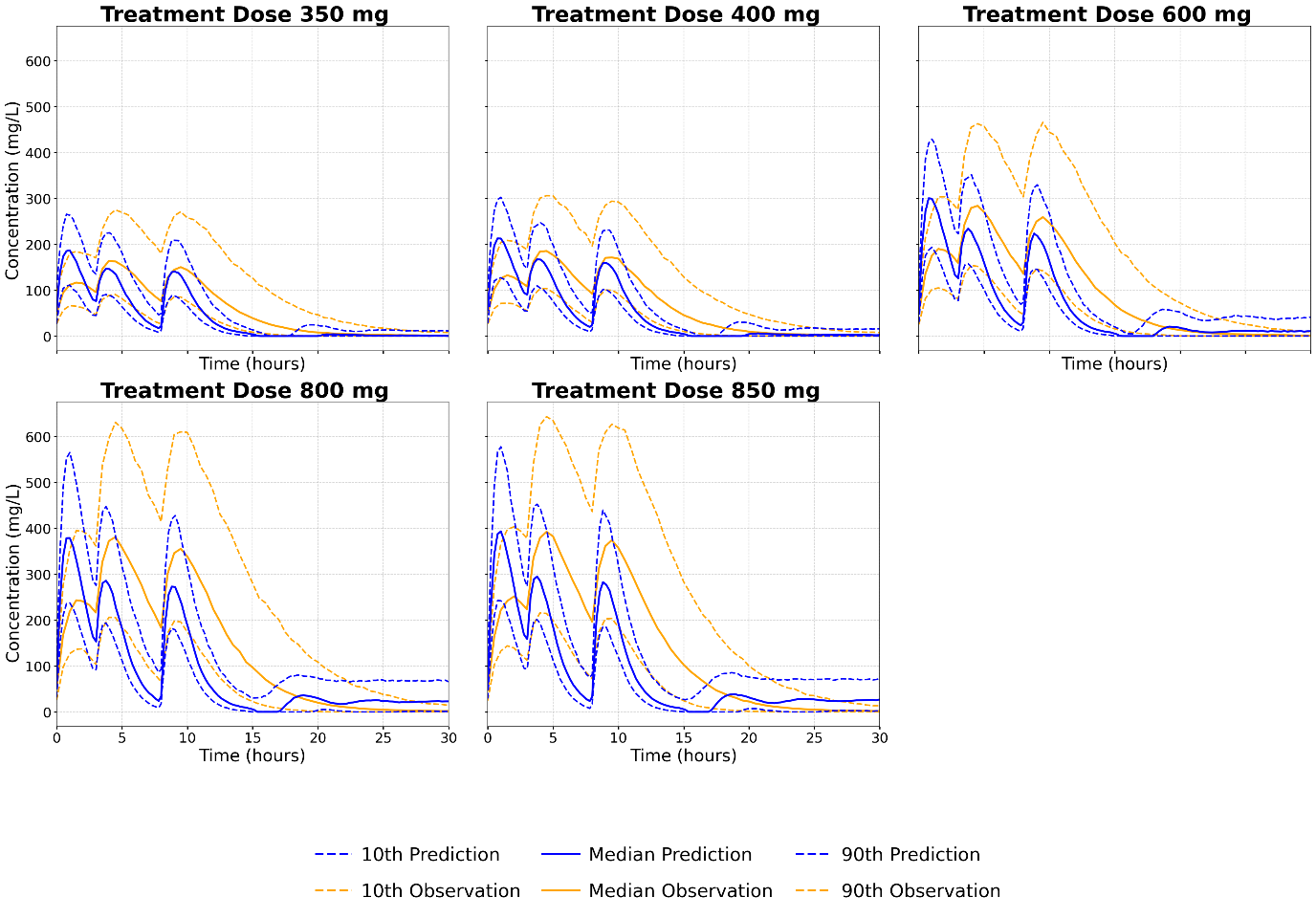


Figure A4. Model reconstruction (blue) evaluation using the variational autoencoder trained, without input-response normalization, on the 400-mg and 800-mg dosing schedules (days 0, 8, and 16), with evaluations performed on dosing regimens of 350, 400, 600, 800, and 850 mg administered on days 0, 3 and 8. Predictions are performed by drawing individual parameters from the posterior from each individual in the test set.
