# Supplementary material for: The Empirical Bayes Variational Autoencoder—A Neural ODE Approach for Population Modeling in Pharmacology
Source: CPT Pharmacometrics Syst Pharmacol. 2026 Jun 17;15(7):e70280. doi: 10.1002/psp4.70280 (PMC13275335; doi:10.1002/psp4.70280)
Supplement: Supplementary file 3 — Data S3: Simulation setup. [file PSP4-15-e70280-s004.docx]

## Simulation setup

The system is described by the following differential equations:

$$\frac{dC_{gut}}{dt}= -k_{a}\cdot C_{gut}$$

$$\frac{dC_{central}}{dt}= k_{a}\cdot C_{gut}-(k_{e}+\beta_{i}x_{i})\cdot C_{central}$$

$$C_{gut}\left( 0 \right)=0, C_{central}\left( 0 \right)=C_{0}$$

where $C_{gut}$ and $C_{central}$ represent the concentration of drug in the gut and central compartments, respectively. IIV was introduced via random effects on the initial concentration ($C_{0}$), absorption rate constant ($k_{a}$), and the elimination rate constant ($k_{e}$). We set the volume of distribution ($V_{d}$) to 1 L without IIV. All other values were sampled from a multivariate normal distribution with a full covariance matrix. Moreover, a potential covariate effect was included on the elimination rate with $\beta_{i}$ being the magnitude of the effect and $x_{i}$ an indicator function denoting if the individual has the covariate or not. Observations were generated using a proportional and additive measurement error model. The initial concentration was not dose-dependent and could be the result of a run-in period prior to the simulated dosing regimen. All parameter values used for the simulation are found in the supplementary information.

Training and validation data were generated using 1000 individuals per treatment group, with drug concentrations sampled every 30 minutes. This large sample size was selected not to mimic a typical pharmacokinetic study, but to ensure stable estimation of population characteristics and to evaluate whether the models could reliably recover the underlying simulated structure. Two dosing schedules were used. The primary schedule—a four-dose regimen applied in both training and one test dataset—involved oral administration at 0, 8, 18, and 24 hours with dose levels of 400 mg or 800 mg. A second test dataset employed a three-dose regimen with dosing at 0, 3, and 8 hours and dose levels of 350, 400, 600, 800, and 850 mg, allowing assessment of both interpolation within observed dosing patterns and extrapolation to unseen doses and intervals. The population PK model used mean parameter values of 0.4 1/h for $k_{a}$, 0.6 1/h for $k_{e}$, 50 mg/L for the baseline concentration, 1 L for $V_{d}$, and 0.3 1/h for the covariate effect on $k_{e}$. Between-subject variability was modeled with standard deviations of 0.5 for $k_{a}$, $k_{e}$, and $C_{0}$, and for the case with correlation, the correlation values were 0.5 between $k_{a}$ and $k_{e}$, 0.35 between $k_{a}$ and $C_{0}$, and 0.2 between $k_{e}$ and $C_{0}$. Residual variability consisted of an additive error of 3 and a proportional error of 0.1.
