# Supplementary material for: The Empirical Bayes Variational Autoencoder—A Neural ODE Approach for Population Modeling in Pharmacology
Source: CPT Pharmacometrics Syst Pharmacol. 2026 Jun 17;15(7):e70280. doi: 10.1002/psp4.70280 (PMC13275335; doi:10.1002/psp4.70280)
Supplement: Supplementary file 4 — Data S4: Results with smaller networks. [file PSP4-15-e70280-s003.docx]

## Results with smaller networks

The results from the experiments in which the models were trained with significantly fewer parameters are shown in Figures A5 and A6, where Figure A5 corresponds to the simulation study and Figure A6 to the theophylline dataset. Figure A5 was selected from all simulation settings because it represents the most challenging setup; diagnostic plots for the other settings were likewise very similar to those presented in the main manuscript.

Figure A5. Empirical Bayes variational autoencoder simulations compared to test data stratified on both treatment arm and covariate. 95% confidence interval, based on 10 simulations, are shown as shaded areas. Trained with smaller network.

Figure A6. Visual predictive check, histogram of individual weighted residuals (IWRES), and observed concentrations plotted against model predictions from an empirical Bayes variational autoencoder trained on the clinical dataset. Trained with smaller network.
